# Disseminated Leiomyomatosis Following a Robotic-Assisted Myomectomy With Power Morcellation: A Case Report

**DOI:** 10.7759/cureus.75971

**Published:** 2024-12-18

**Authors:** Christopher Whalen, Moira Murphy, Patrick Timmins

**Affiliations:** 1 School of Medicine, Touro College of Osteopathic Medicine, Middletown, USA; 2 Obstetrics and Gynecology, Vassar Brothers Medical Center, Poughkeepsie, USA

**Keywords:** disseminated peritoneal leiomyomatosis, fibroids, leiomyomas, power morcellator, sydney in bag morcellation technique, uterine leiomyomata

## Abstract

This case reports a 44-year-old female who presented to the gynecologic oncology clinic status post robotic-assisted laparoscopic myomectomy with intraperitoneal unprotected power morcellation in 2012, with an incidental finding of three conglomerate solid masses in the abdomen above the uterus, with each mass measuring approximately 15.5 cm. The patient underwent an exploratory laparotomy where multiple masses greater than 10 cm were found scattered throughout the abdominal cavity. The masses were resected, and the patient underwent an appendectomy, bilateral salpingectomy, as well as a total abdominal hysterectomy and small bowel resection. Pathology reports revealed benign leiomyomas. The patient’s presentation was consistent with disseminated peritoneal leiomyomatosis in the setting of previous myomectomy with the use of power morcellation. Disseminated peritoneal leiomyomatosis is exceedingly rare, and upon encountering this case, further research was conducted to explore the complications associated with the use of power morcellators in gynecological procedures.

## Introduction

Uterine leiomyomata, or fibroids, are one of the many benign tumors that can occur in female patients, being present in about one in every four women [1]. Leiomyomata are noncancerous growths of the uterine tissue and smooth muscle that mainly affect individuals in their reproductive years. The exact etiology of uterine leiomyomata remains unknown, but continued research has suggested potential genetic contributions. Cytogenetic analysis of uterine fibroids has found chromosomal abnormalities across chromosomes three, six, seven, 13, trisomy 12, reciprocal translocation between chromosomes 12 and 14, and monosomy 22 [2]. A relative of a family member with uterine fibroids was also found to be at an increased risk of being afflicted with this condition [3]. A threefold greater incidence of uterine fibroids was seen amongst African American females when compared to other ethnicities, and an occurrence of fibroids in 70% of White women and 80% of African American women [4,5]. 

While this condition is very prevalent in the general population, many of those afflicted are not aware of their diagnosis as it is often asymptomatic. The development of symptoms from this pathology depends on the size, location, and number of fibroids present. These factors' summative impact leads to mass-effect symptoms, such as dysmenorrhea, menorrhagia, and dyspareunia. Patients may also present with chronic pelvic pressure, constipation, and frequent urination. Symptomatic presentation occurs in only approximately 30% of patients with uterine fibroids [4]. If the patient is asymptomatic, treatment is not necessary.

The most common initial screening tool for diagnosing uterine leiomyomata is a transabdominal or transvaginal ultrasound, as it is the least invasive and lowest-risk modality that can provide accurate diagnostic information. Commonly used medical management includes a GnRH agonist to shrink fibroids by inhibiting the release of gonadotropic hormones from the anterior pituitary through desensitization of the GnRH receptors on the gonadotropic cells of the anterior pituitary [6]. Alternatively, a progesterone intrauterine device has also been helpful for symptomatic management of fibroids by decreasing bleeding through stabilization of the endometrium with local progesterone release [7]. While these conservative treatments are commonly used, patients do have the option of various invasive procedures, including myomectomy, hysterectomy, radiofrequency ablation, endometrial ablation, and uterine artery embolization for more definitive treatment [3]. 

Myomectomy is the most common surgery in the management of uterine leiomyomata and involves direct resection of the leiomyomas. It is often the surgery of choice in women who wish to preserve their fertility. In the setting of large uterine fibroids or an enlarged fibroid uterus, the use of a power morcellator is often required. This tool is used in conjunction with laparoscopic surgery and allows large specimens to be removed from the surgical port sites by mechanical mincing of the specimen into smaller pieces by the power morcellator. Although the use of a morcellator preserves the ability to continue with laparoscopic surgery, this tool has potential risks and rare adverse effects. The main risk associated with the use of a morcellator is the dissemination of particles and possibly spreading undiagnosed uterine cancer by virtue of the morcellator dissembling large tumors prior to removal from the surgical port in a non-contained manner. In this case, we explore the potential dissemination of leiomyoma across the peritoneal cavity post-laparoscopic myometry with the use of a power morcellator. 

## Case presentation

We herein present a case involving a 44-year-old female G6P3 with a past medical history of hypertension controlled with medication, obesity, sexually transmitted diseases including herpes simplex virus (HSV) and human papillomavirus (HPV), and pelvic inflammatory disease who was referred to the gynecologic oncology department for asymptomatic pelvic masses.

**Figure 1:**
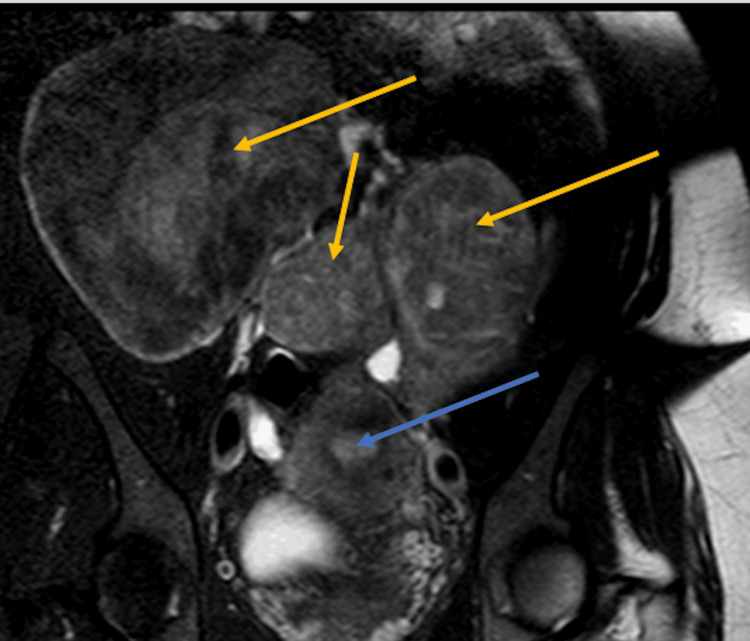
Coronal MRI of the lower abdomen. Blue Arrow: Points to the endometrium of the uterus; Yellow Arrows: Point to the inta-abdominal masses.

In 2012, the patient originally presented to her obstetric-gynecologist (OB/GYN) with menometrorrhagia and large uterine fibroids, as well as intramural leiomyoma of the uterus. The patient underwent an uncomplicated robotic-assisted myomectomy with morcellation without post-surgical complications. In the fall of 2022, she presented to her primary care doctor for bariatric surgery evaluation. The patient received a pelvic sonogram in September 2022, which showed three conglomerate solid masses superior to the uterus, with each mass measuring approximately 15.5 cm. After this finding, a follow-up MRI was ordered, which revealed multiple intra-abdominal masses but no lymphadenopathy or ascites (Figure 1). At this time, sarcomatous degeneration could not be ruled out. The patient denied any family history of uterine, breast, or ovarian cancer.

Objectively, the patient’s vital signs were within normal limits, excluding her BMI of 42.8. On a comprehensive physical exam, the patient was found to have a nontender abdomen with palpable intra-abdominal masses, including one at the uterine fundus. This patient was without appreciable lymphadenopathy. Genitourinary physical exam was limited by patient body habitus, but no abnormalities or pathology were noted on examination of the external genitalia, vaginal vault, or cervix. 

Based on the subjective and objective findings, including the diagnostic imaging, the intra-abdominal masses were suspected to be leiomyomata. However, all diagnostic possibilities were discussed with the patient, including any benign, atypical, and malignant possibilities, and it was indicated that a further workup with pathology would be necessary to confirm. Nonsurgical and surgical options were discussed with the patient, and she endorsed her preference of removing what was necessary but would like to preserve her ovaries if possible to prevent early-onset menopause. The risks and benefits of the surgery were discussed with the patient, and she was scheduled for an exploratory laparotomy for multiple intra-abdominal masses.

During the surgery, multiple masses greater than 10 cm were scattered throughout the abdominal cavity and attached to the small bowel, bladder, omentum, sigmoid mesentery, appendix, small bowel mesentery, abdominal wall, and pelvic peritoneum. All visible masses were resected, and the patient underwent an appendectomy, bilateral salpingectomy, as well as a total abdominal hysterectomy. The ovaries were not involved with the extensive disease and were left in vivo as per the patient’s request. The surgery was complicated by the firm attachment of the small bowel mass and subsequent formation of a friable bowel, requiring evaluation by gastrointestinal oncology. A small bowel resection was done after resection of the mass due to complications from mass adherence. All masses were sent to frozen pathology for evaluation, and the reports indicated benign leiomyoma. After the surgical procedures, the abdomen was scanned, and no residual disease was noted. Postsurgical evaluation was consistent with disseminated peritoneal leiomyomatosis. The patient had a three-day postoperative hospital course with no complications and was discharged on the third day. She was recommended to follow-up with the OB/GYN oncologist, and is currently doing well.

## Discussion

The use of a power morcellator in general surgery, urology, and gynecology began in the 1990s [8]. The power morcellator was introduced as a surgical tool, helpful in breaking up large masses into finer components that are more easily removed through a surgical port. This tool was enlisted in an effort to continue performing laparoscopic surgery and avoiding conversion to higher-risk open surgeries. However, the power morcellator comes with its own set of risks and disadvantages. In gynecologic surgeries, such as hysterectomy or myomectomy, the use of a power morcellator has been successful in removing large uteruses and fibroids but has placed the patients at a higher risk for rare complications, including intravenous leiomyomatosis, benign metastasizing leiomyoma, parasitic leiomyoma, and disseminated peritoneal leiomyomatosis. 

In 2021, Liu reported that, to date, there were approximately 200 cases of disseminated peritoneal leiomyomatosis reported in the literature [9]. Reported cases of this complication have been due to the seeding of uterine fibroid tissue within the abdominal cavity and pelvis following myomectomy.

Benign metastasizing leiomyoma is another rare complication, with less than 150 cases reported in the literature, that is named after its tendency to present with leiomyomas at sites distant from the uterus [10]. Literature suggests that during the surgery, there is seeding of the abdominal cavity with leiomyoma that is capable of spreading hematogenously or lymphatically [11]. However, there were a surprising number of cases in which there were no previous surgeries, but individuals still presented with this disease. In these cases, coelomic metaplasia could possibly explain these patients' disease processes. Coelomic metaplasia suggests that the mesothelium has the potential to undergo metaplastic changes in the presence of estrogen, which can lead to the proliferation of endometrial stroma and fibroid formation outside the uterus [12]. Symptomatology is dependent on the location of metastasis, with the lung being most commonly involved. Management of this disease is complex, and the focus is to subdue the hormonal stimulation of these lesions to prevent further growth, whether performed medically or surgically [8]. 

Intravenous leiomyomatosis is a third rare complication of power morcellator use. Currently, there are 300 cases reported in the literature [13]. More than 50% of cases are associated with a previous hysterectomy before the onset of the disease [14]. The pathophysiology of this disease is not completely understood but is hypothesized to be due to the extension of the leiomyoma into the ovarian veins that subsequently drain into the inferior vena cava. These patients can present with the usual mass symptoms of leiomyoma, but intravenous extension of the mass can also occlude the vein, causing palpitations and chest tightness.

Lastly, in 2017, there have been less than 30 reported cases of parasitic leiomyoma [15]. This disease is also thought to be from simple seeding of endometrial fibroids within the abdominal cavity following the use of a power morcellator. This disease got its name from leiomyomas utilizing new blood wherever they land in the abdominal cavity and no longer feeding on their previous source, similar to parasites exploiting various hosts for favorable resources. Management of this disease involves resection, but caution must be taken as there is a possibility that the leiomyoma formed a new complex blood supply [16].

In the case of this patient, she had multiple greater than 10 cm masses removed from the abdominal cavity, most likely from her previous myomectomy with morcellation. A case report by Dr. Liu in 2022 describes a patient who also had a previous laparoscopic myomectomy and returned with a diagnosis of disseminated peritoneal leiomyomatosis [9]. This report, when compared to other similar case reports, suggests an increased risk of tissue seeding into the abdominal cavity during mass removal. It was reported that the likelihood of disseminated peritoneal leiomyomatosis following laparoscopic myomectomy was 0.12% to 0.95% [17]. Given the current literature, history, and surgical findings, this patient has features consistent with disseminated peritoneal leiomyomatosis as a complication of her previous myomectomy with the use of a power morcellator. 

Based on these complications and adverse events, the Food and Drug Administration (FDA) issued a warning regarding the use of power morcellators in patients undergoing surgery for uterine fibroids. They estimated that there may be a hidden uterine sarcoma present in approximately one in 225 to one in 580 patients electing to undergo surgery for uterine fibroids, and an undiagnosed leiomyosarcoma may be present in one in 495 to one in 1,1000 patients [18]. The FDA also set forth recommendations that a patient who is postmenopausal or older than 50 years of age should not undergo surgery with laparoscopic power morcellators for the removal of uterine tissue, as the risk of spreading undetected cancer is too great [18]. 

There have been various attempts to mitigate the complications and side effects of the power morcellator in various intra-abdominal surgeries. The most notable is the introduction of a bag in conjunction with the use of a power morcellator. In 2014, the “Sydney in bag morcellation technique” was described by Einarsson as a method for containing abdominal morcellation in multiport laparoscopic surgery [19]. This technique uses a 15-mm EndoCatch bag and an Ancho TRS-200 tissue retrieval system to complete this procedure. The specimen is placed inside the retrieval bag and power morcellation is performed under optic visualization in an insufflated abdomen. The morcellation remnants were then removed from the abdomen within the bag, thus decreasing the risk of spread. Recently, in February 2020, the FDA released an updated Safety Communication with recommendations on the use of laparoscopic morcellation for myomectomy or hysterectomy. They indicated that this procedure should only be performed with a tissue containment system legal in the United States or one that is specifically designed for laparoscopic power morcellators [18, 20]. The “Sydney in bag morcellation technique” is an attempt to mitigate the complications of power morcellator use and one that the FDA deemed appropriate.

## Conclusions

This case can be used to highlight various rare complications of myomectomy in conjunction with power morcellator use. The current literature contains limited cases similar to this patient, but such cases involved procedures that included the use of a morcellator. Following the development of such complications, the FDA put forth a statement regarding the use of a sterile tissue contaminant device to decrease this risk. Given the patient’s history and current literature, this case may have been prevented if a tissue contaminant device had been used with the power morcellator for the removal of her leiomyomas in 2012. Going forward, for myomectomies and gynecologic surgeries involving endometrial tissue, surgical teams must be aware and cautious when resecting a mass with the use of a power morcellator. As recommended by the FDA, power morcellation should be performed only with a tissue containment device to prevent such cases from developing in the future. This case involving disseminated peritoneal leiomyomatosis can serve as an example of a rare complication of uncontained power morcellator use in gynecologic surgery. We hope our case can be used to highlight the various techniques developed to prevent these rare complications from occurring in the future and as a reminder of the importance of proper surgical techniques to prevent complications and adverse patient outcomes.
